# Transcriptome analyses reveal the flowering regulatory networks in the desert ephemeral plant *Eremopyrum triticeum*

**DOI:** 10.3389/fpls.2025.1576519

**Published:** 2025-05-08

**Authors:** Ling-Lu Zeng, Wan-Qiu Meng, Xue-Ni Zhong, Jun-Jie Peng, Xiu-Li Yang, Li Sun

**Affiliations:** College of Life Sciences, Shihezi University, Shihezi, Xinjiang, China

**Keywords:** *Eremopyrum triticeum*, transcriptome, flowering pathway, differentially expressed genes, flowering locus T

## Abstract

*Eremopyrum triticeum*, an annual spring ephemeral plant and a wild relative of wheat (*Triticum aestivum*), is widely distributed in the Junggar Desert of northern Xinjiang, China. It has several adaptive traits to survive in the desert environments, such as rapid growth in the early spring, flowers quickly, and completes its life cycle within approximately two months. However, the adaptation mechanisms of the fast flowering are still unknown. In this study, high-throughput RNA sequencing (RNA-seq) was performed to identify differentially expressed genes (DEGs) associated with flowering in *E. triticeum* during three developmental stages. A total of 11,278 DEGs were identified, including 1,632 DEGs specifically expressed during the flowering stage. Pathway analysis showed that these DEGs are mainly enriched in plant-pathogen interaction, plant hormone signal transduction, the MAPK signaling pathway, and so on. A total of 92 DEGs related to the flowering pathway were identified, which are mainly involved in the photoperiod, hormone signaling, autonomous, and vernalization pathway. Multiple transcription factor families related to floral transition were identified, with members of the MADS-box, bHLH, MYB, and AP2 families being the most abundant. In addition, four *FLOWERING LOCUS T* (*FT*) genes were identified in *E. triticeum*, and three of them were highly up-regulated at the flowering stage. The expression of *EtFT-1* was induced in darkness, and short-day conditions promote its expression. Overexpression of the *EtFT-1* gene accelerates flowering in *Arabidopsis*.

## Introduction

Spring ephemeral plants are a significant component of desert flora. They are able to use winter snowmelt and early spring rainfall to germinate, grow and flower rapidly, and complete their life cycle in two to three months (Wang, 1993; Zhang and Chen, 2002). Spring ephemeral plants are an important part of the flora of the Gurbantunggut desert in Northern Xinjiang, China (Mao and Zhang, 1994). In the month of May, their cover here can be as high as 40%, making them significant for dune stabilization and environmental sustainability during the spring season (Wang et al., 2003). These species have developed specialized adaptive mechanisms that enable them to be effective in the harsh desert environment, including rapid growth and flowering, high photosynthesis ability under high light intensity conditions, high seed production potential, and resistance against abiotic stress factors. Understanding the adaptation mechanisms of ephemeral plants helps to elucidate the survival strategies of plants in extreme desert environments and provides important clues for ecological adaptation and evolutionary studies. Furthermore, these plants play an important role in desert ecosystems by stabilizing sand dunes and maintaining the ecological balance in spring arid environments, which is important for combating desertification and protecting biodiversity.

*Eremopyrum triticeum* (Gaertn.) Nevski (2n = 2x = 14) is an annual spring ephemeral plant and one of the wild relatives of wheat (*Triticum aestivum*) (Frederiksen and Bothmer, 1995). In China, it is mainly distributed in the southern part of Junggar Desert and the Ili Valley (Zhang, 2014), where it serves as an important component of desert and steppe grassland ecosystems. This species plays an important ecological role in stabilizing soil, maintaining biodiversity, and providing an essential forage resource in early spring (Wang et al., 2003). To cope with the harsh desert environment, *E. triticeum* has evolved a number of specialized adaptive traits, including rapid germination, growth, and flowering, which allow it to efficiently utilize winter snowmelt and early spring rainfall. It completes its life cycle in about two months, from late March to early April, before the onset of extreme summer drought (Zhang, 2014; Qiu et al., 2007). In addition, *E. triticeum* exhibits heterochronic germination, with seeds capable of germinating in both spring and fall, allowing it to take advantage of favorable conditions across seasons and increasing its adaptability to desert environments (Wang et al., 2024). The species exhibits high photosynthetic efficiency under intense sunlight, seed yield, low temperature tolerance, drought and salinity resistance, allowing it to survive and reproduce in extreme desert environments (Zhang, 2014). Despite these ecological and adaptive advantages, the molecular mechanisms underlying its rapid flowering and environmental resistance remain largely unknown.

The flowering transition is a critical process in the life cycle of a plant, as it marks the transition from vegetative growth to reproductive growth. The timing of flowering is regulated by both internal genetic elements and external environmental signals, including photoperiod, temperature change, and stress (Kurokura et al., 2013). In *Arabidopsis*, six pathways have been identified that regulate flowering: the photoperiod pathway, the gibberellin (GA) pathway, the vernalization pathway, the environmental temperature pathway, the aging pathway, and the autonomous pathway (Fornara et al., 2010; Srikanth and Schmid, 2011; Kinoshita and Richter, 2020). These different pathways are coordinated into a regulatory network controlled by a group of key regulators, including FLOWERING LOCUS T (FT), GIGANTEA (GI), SUPPRESSOR OF OVEREXPRESSION OF CONSTANS 1 (SOC1), FLOWERING LOCUS C (FLC), TWIN SISTER OF FT (TSF), and LEAFY (LFY) (Parcy, 2005; Moon et al., 2005). Among these regulators, FT is the key regulator controlling the floral transition (Suárez-López et al., 2001). FT is a member of the phosphatidylethanolamine binding protein (PEBP) superfamily that functions as a mobile floral signal. It plays a critical role in the regulation of flowering time. The PBP family in *Arabidopsis* contains six members that are divided into three subfamilies: TFL1-like, FT-like, and MFT-like. The MFT-like subfamily is the ancestor of the FT-like and TFL1-like subfamilies (Liu et al., 2016). FT expression is controlled by CONSTANS (CO), a member of the CONSTANS-LIKE (COL) family of B-box zinc finger transcription factors. SOC1, a MADS box transcription factor, is a floral activator that regulates flowering time, floral patterning, and meristem identity (Lee and Lee, 2010). Conversely, FLC, another MADS box transcription factor, is a key repressor of flowering through repression of FT and SOC1 expression (Michaels and Amasino, 1999; Helliwell et al., 2006). Activation of floral promoters subsequently induces the expression of floral meristem identity genes such as *LFY* and *APETALA 1* (*AP1*), which ultimately initiates the flowering process (Blázquez et al., 1997).

In the *Arabidopsis* photoperiod pathway, FT and CO are essential promoters of flowering, whereas in cereals such as wheat, the vernalization pathway is regulated by genes such as *VRN1*, *VRN2*, *VRN3*, and *VRN4* (Yan et al., 2003, 2004, 2006; Kippes et al., 2015). In the autonomous pathway, factors such as FLOWERING LOCUS D (FLD), LUMINIDEPENDENS (LD), FLOWERING LOCUS PA (FPA), FLOWERING LOCUS CA (FCA), FLOWERING LOCUS VE (FVE), and FLOWERING LOCUS Y (FY) facilitate flowering by repressing *FLC* expression (Michaels and Amasino, 2001).

The age pathway is primarily regulated by microRNA156 (miR156), targets the SQUAMOSA-PROMOTER BINDING LIKE (SPL) transcription factors, and are essential for the regulation of flowering in *A. thaliana* (Zheng et al., 2019). In addition to GA, other endogenous hormones, such as auxins, abscisic acid (ABA), salicylic acid (SA), jasmonic acid (JA), ethylene, and brassinosteroid (BR) also play positive or negative roles in the regulatory network of flowering (Mutasa-Göttgens and Hedden, 2009; Davis, 2009; Diezel et al., 2011; Campos-Rivero et al., 2017).

Flowering transition is also regulated by environmental factors such as day and night length, drought, salinity, cold, and heat (Kazan and Lyons, 2016; Takeno, 2016; Lee et al., 2023). In response to drought stress, plants tend to accelerate flowering, a process called drought escape (Sherrard and Maherali, 2006; Franks et al., 2007; Bernal et al., 2011). The photoperiodic flowering pathway is closely related to drought-induced flowering regulation. In *Arabidopsis*, drought stress promotes early flowering under long days by activating *FT* gene expression, whereas it delays flowering transition under short days by increasing *FLC* gene expression. In contrast, salinity stress usually delays flowering (Kim et al., 2007), and several key flowering regulators involved in this response have been identified (Ryu et al., 2014; Ma et al., 2015).

To investigate the molecular mechanisms underlying rapid flowering of *E. triticeum* in desert environments, and to identify key genes and regulatory networks involved in flowering time control, RNA-seq analyses were performed at three developmental stages. Potential candidate differentially expressed genes (DEGs) involved in flowering regulation were identified and their functional properties were investigated to understand the molecular mechanism of early flowering in *E. triticeum*. In addition, the expression patterns and ectopic expression of the *EtFT-1* gene were examined to explore its role in flowering time regulation. This study represents the first attempt to elucidate the genetic regulation of flowering in *E. triticeum* and provides insights into the molecular regulatory mechanism of flowering that might be shared with the other spring ephemerals.

## Materials and methods

### Plant material and treatment

In 2021, *E. triticeum* seeds were sown in the experimental field at Shihezi University, Xinjiang, China (43°26′N, 84°58′E). Tissue samples were collected for RNA sequencing at three different developmental stages: vegetative stage (VS), heading stage (HS), and flowering stage (FS). At the VS stage, 12 days after germination, leaves were collected as controls. Spikelets were collected at the HS stage, which occurred 42 days after germination, when they were just heading, and at the FS stage, which occurred 67 days after germination. Ten *E. triticeum* plants per sample were used for each developmental stage. In particular: Four leaves from each plant were gathered at the vegetative stage (VS). A single spikelet from each plant was gathered at the heading and flowering stages. Three distinct biological replicates yielded a total of nine samples for transcriptome sequencing. All of the samples were immediately frozen in liquid nitrogen and stored at -80°C.

For diurnal expression analysis, *E. triticeum* seeds were sown in a pre-mixed soil substrate (nutrient soil:vermiculite:perlite = 3:1:1) and grown in a growth chamber under long-day (LD, 16 h light/8 h dark) or short-day (SD, 8 h light/16 h dark) conditions, with temperatures ranging from 18-22°C and relative humidity between 40% and 60%. After three weeks, young leaves were collected every 4 h for a period of 24 h, starting from time 0 (ZT0) in both LD and SD photoperiods. Collection was completed within 48 h, and leaf samples were collected at the end of each photoperiod. Five leaves from young plants were collected per sample, with three biological replicates for each condition. All samples were immediately snap frozen in liquid nitrogen and stored at -80°C until further analysis.

### Illumina sequencing and gene functional annotation

1 µg of RNA was used for RNA sample preparation. Sequencing libraries were prepared using the NEBNext^®^ Ultra™ RNA Library Prep Kit for Illumina^®^ (NEB, USA) according to the manufacturer’s guidelines, and index codes were added to associate sequences with each sample. Briefly, mRNA was purified from total RNA using poly-T oligo-attached magnetic beads. Fragmentation was performed with divalent cations at high temperature in NEBNext First Strand Synthesis Reaction Buffer (5X). First-strand cDNA was synthesized using random hexamer primers and M-MuLV reverse transcriptase. Second-strand cDNA was synthesized using DNA Polymerase I and RNase H. PCR was performed using Phusion High - Fidelity DNA Polymerase, Universal PCR Primers and Index (X) Primer. Finally, the PCR products were purified (AMPure XP system) and the library quality was evaluated on the Agilent Bioanalyzer 2100 system. Transcriptome sequencing was performed on an Illumina HiSeq 2000 platform with three independent biological replicates. The RNA-sequencing was performed by Biomarker Technologies (Beijing, China).

The sequences were further processed with a bioinformatic pipeline tool, BMKCloud (www.biocloud.net) online platform. To ensure high quality clean data, adapter sequences, poly-N sequences and low-quality reads were removed from the raw data. Subsequently, the Q20, Q30, GC content and sequence duplication levels of the clean data were calculated to assess the quality of the sequencing. *De novo* assembly of the transcriptome was performed using Trinity software (Grabherr et al., 2011). Using DIAMOND software (Buchfink et al., 2015), unigene sequences were compared to several databases, such as NR, Swiss-Prot, COG, KOG, eggNOG4.5, and KEGG. KOBAS (Xie et al., 2011) was used to get KEGG orthology data. The sequences were analyzed using InterProScan (Jones et al., 2014), a program that combines information from several databases, to produce Gene Ontology (GO) annotations. Additionally, HMMER software (Eddy, 1998) was used to compare unigene amino acid sequences to the Pfam database in order to identify functional domains.

### Differential expression analysis, gene ontology and KEGG pathway enrichment analysis

Expression levels were quantified using the fragments per kilobase per million reads (FPKM) method (Trapnell et al., 2010). Differential expression between groups was analyzed using the DESeq2 package (Love et al., 2014). To control for false positives, we applied the Benjamini-Hochberg method to adjust *p*-values, resulting in a false discovery rate (FDR). Differentially expressed genes were identified using the criteria of |log2 fold change (FC)| ≥ 1 and FDR < 0.01. FC represents the expression ratio between the two groups.

Gene Ontology (GO) enrichment analysis of the DEGs was performed using the R package topGO based on the Kolmogorov-Smirnov test. The KEGG database provides high-level information on the functions and utilities of biological systems (Kanehisa et al., 2004). KOBAS software was used to assess the statistical enrichment of DEGs in KEGG pathways (Xie et al., 2011).

### Phylogenetic analysis

Protein sequences were obtained from the NCBI database. Homologous sequences were aligned using CLUSTALW, and a phylogenetic tree was constructed using MEGA 7.0 software with the neighbor-joining (NJ) method, with 1000 bootstrap replicates.

### Quantitative real-time PCR analysis

Total RNA was isolated from each sample according to the protocol provided with the RNA-prep Pure Plant Kit (TIANGEN Biotech, China). Then, 1 µg of total RNA was used to synthesize first-strand cDNA using the PrimeScript RT Reagent Kit (Takara, DRR037A). To verify the reliability of the gene expression profiles from the RNA-seq data, nine genes, including *EtFT-1* (c164910.graph_c0), *EtFKF1* (c162671.graph_c3), *EtPP2C* (c149628.graph_c0), *EtLHY* (c162145.graph_c0), *Et*CO (c160085.graph_c2), *EtPIF4* (c162394.graph_c2), *Et*GID1 (c161985.graph_c0), *Et*RGL1 (c81081.graph_c0), and *Et*BRI1 (c154802.graph_c1), involved in flowering regulation and hormone signaling, were selected for qRT-PCR on an ABI 7500 Fast real-time PCR system (Applied Biosystems, USA) using ChamQ Universal SYBR qPCR Master Mix (Vazyme, Nanjing, China). The qRT-PCR validation analyses were performed on the same nine samples as the RNA-seq analyses, with three technical replicates for each sample. Relative expression levels were evaluated using the 2^-ΔΔCt^ method (Livak and Schmittgen, 2001). The 18S rRNA gene of *E. triticeum* (GenBank accession no. PQ072787) was used as an internal control. The reference treatment was vegetative stage (VS). The results of the statistical analysis were expressed as the mean ± standard deviation of the three replicates. Furthermore, the circadian expression level of *EtFT-1* was assessed by qRT-PCR analysis, and the 2^−ΔCt^ technique was used to quantify relative expression. The 18S rRNA gene of *E. triticeum* was served as an internal control. The specific PCR primers are listed in **Supplementary Table 9**.

### Generation of *EtFT-1* transgenic *Arabidopsis*

The open reading frame (ORF) of *EtFT-1* was inserted into the plant expression vector pCAMBIA2300 at the *Kpn* I and *Xba* I restriction sites to construct the recombinant plasmid 35S::*EtFT-1* under the control of the CaMV 35S promoter. The recombinant vector and empty pCAMBIA2300 vector were then transferred into *Agrobacterium tumefaciens* strain GV3101 by heat shock treatment. The validated recombinant vector was transformed into *Arabidopsis* (Col-0) by the floral dipping method (Clough and Bent, 1998). Homozygous transgenic *Arabidopsis* lines were selected on MS medium supplemented with 50 mg/L kanamycin. The overexpressing T3 transgenic lines were collected for subsequent experiments. Four weeks after planting, samples of wild-type (WT) and transgenic lines were collected for total RNA extraction and reverse transcription-polymerase chain reaction (RT-PCR) to analyze the expression levels of *EtFT-1*. The specific PCR primers are listed in **Supplementary Table 9**.

### Data analysis

All experimental data were analyzed using SPSS 26.0 software. A one-way ANOVA was first performed, followed by a *t*-test and Duncan’s multiple comparisons to assess differences between groups. Statistical significance was set at *p* < 0.05 and *p* < 0.01, respectively.

## Results

### Sequencing, assembly and functional annotation

**Figure 1:**
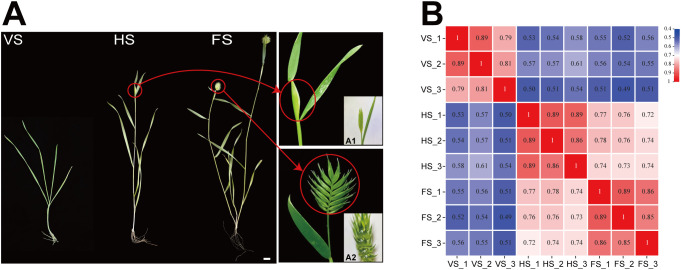
Three developmental stages of the vegetative growth and flowering stages of *E*. *triticeum* **(A)** and Pearson correlation coefficients of nine samples **(B)**. A1. The spikelet at the heading stage under a stereomicroscope; A2. Lateral view of the spikelet at the flowering stage under a stereomicroscope. Arrows point to the florets. Scale bar: 10 mm. VS, vegetative growth stage; HS, heading stage; FS, flowering stage.

A total of nine samples from three developmental stages (VS, HS, and FS) were analyzed (**Figure 1A**), yielding 193,417,047 clean reads (equivalent to 57.78 GB) after removing adapter sequences, contaminants, and low-quality reads. The average Q30 and GC content was 94.71% and 53.35%, respectively. A total of 86,950 transcripts and 41,289 unigenes were identified from these contigs, with an average length of 1,149 base pairs and an N50 of 1,875 base pairs (**Supplementary Table 1**, **Supplementary Table 2**). To assess gene expression differences between pairs of samples, Pearson correlation coefficients were calculated and the correlation values for all nine datasets (41,289 unigenes in total) were visualized in a heatmap (**Figure 1B**). The results indicated that the three biological replicates at each time point showed a high degree of correlation.

Functional annotation was performed against several publicly available nucleotide and protein databases. Homologs of the 41,289 unigenes were identified in the following databases with an E-value cutoff of 1e-5 (**Supplementary Table 3**): COG (7,573 unigenes, 18.34%), GO (21,179 unigenes, 51.29%), KEGG (16,067 unigenes, 38.91%), KOG (14,943 unigenes, 36.19%), Pfam (19,572 unigenes, 47. 40%), Swiss-Prot (15,576 unigenes, 37.72%), TrEMBL (26,295 unigenes, 63.69%), EggNOG (20,411 unigenes, 49.43%), and NR (26,545 unigenes, 64.29%). After eliminating redundancies from different databases, 28,956 unigenes were annotated at least once, representing about 70.13% of the total. Statistical comparison of the distributed E-values revealed that a remarkable 78.54% of the mapped sequences had high homology (E-value < 1e-30) and 60.22% had very high homology (E-value < 1e-50) when compared with plant sequences annotated in the NR database (**Supplementary Figure 1A**). Similarity distribution analysis showed that 85.09% of the unigenes had a similarity level of at least 60%, and 53.31% of the unigenes had a similarity level between 90% and 100% (**Supplementary Figure 1B**). Among the genes annotated in the NR database, the highest percentage of matches was associated with *Aegilops tauschii* (28.11%), *Triticum turgidum* (23.39%), and *Hordeum vulgare* (15.85%) (**Supplementary Figure 1C**).

### Differentially expressed genes identification

**Figure 2:**
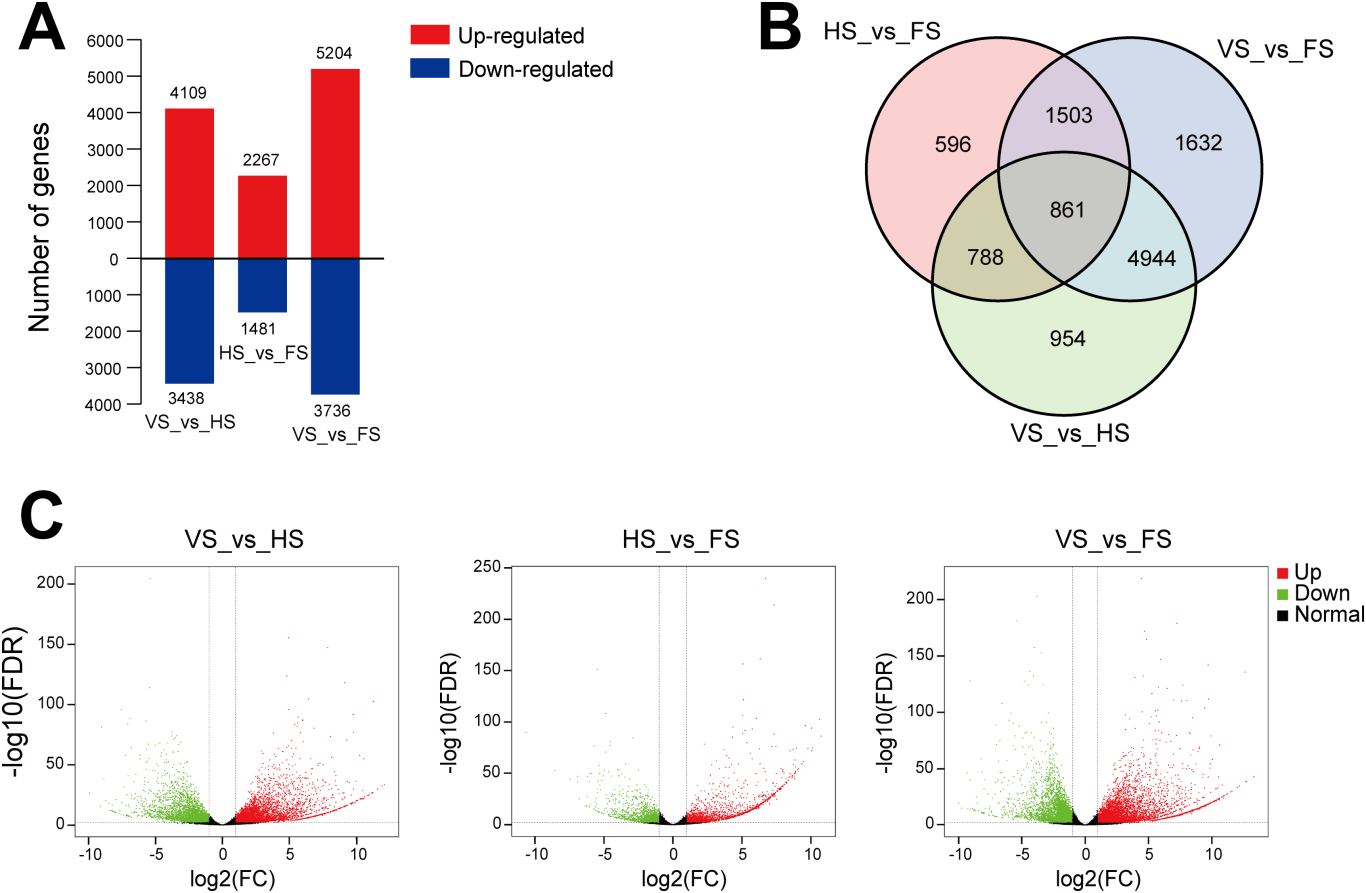
Transcriptome profiling of *E*. *triticeum* at three different developmental stages. **(A)** DEGs identified from different comparisons. **(B)** Venn diagram of DEGs at the three developmental stages. **(C)** Volcano plot of DEGs. Red and green dots indicate up- and down-regulated DEGs, respectively, and black dots indicate genes with no significant differences. VS, vegetative stage; HS, heading stage; FS, flowering stage.

To identify the DEGs related to flowering, we analyzed the transcript levels of each unigene at three developmental stages, and the DEGs were defined by a log2 (fold change) ≥ 1 and a false discovery rate (FDR) ≤ 0.01 (**Figure 2**). A total of 11,278 DEGs were identified in the *E. triticeum* RNA-seq data. Among them, 3,748 DEGs were found to vary in HS_vs_FS (2,267 up-regulated and 1,481 down-regulated) (**Figure 2A**). Venn diagram analysis revealed that 596 transcripts were differentially expressed in the flowering stage compared to the heading stage but not differentially expressed over the other comparisons of developmental stages (**Figure 2B**). In the volcano plot, the number of up-regulated DEGs was more significant than the number of down-regulated DEGs in VS_vs_HS, HS_vs_FS and VS_vs_FS (**Figure 2C**).

**Figure 3:**
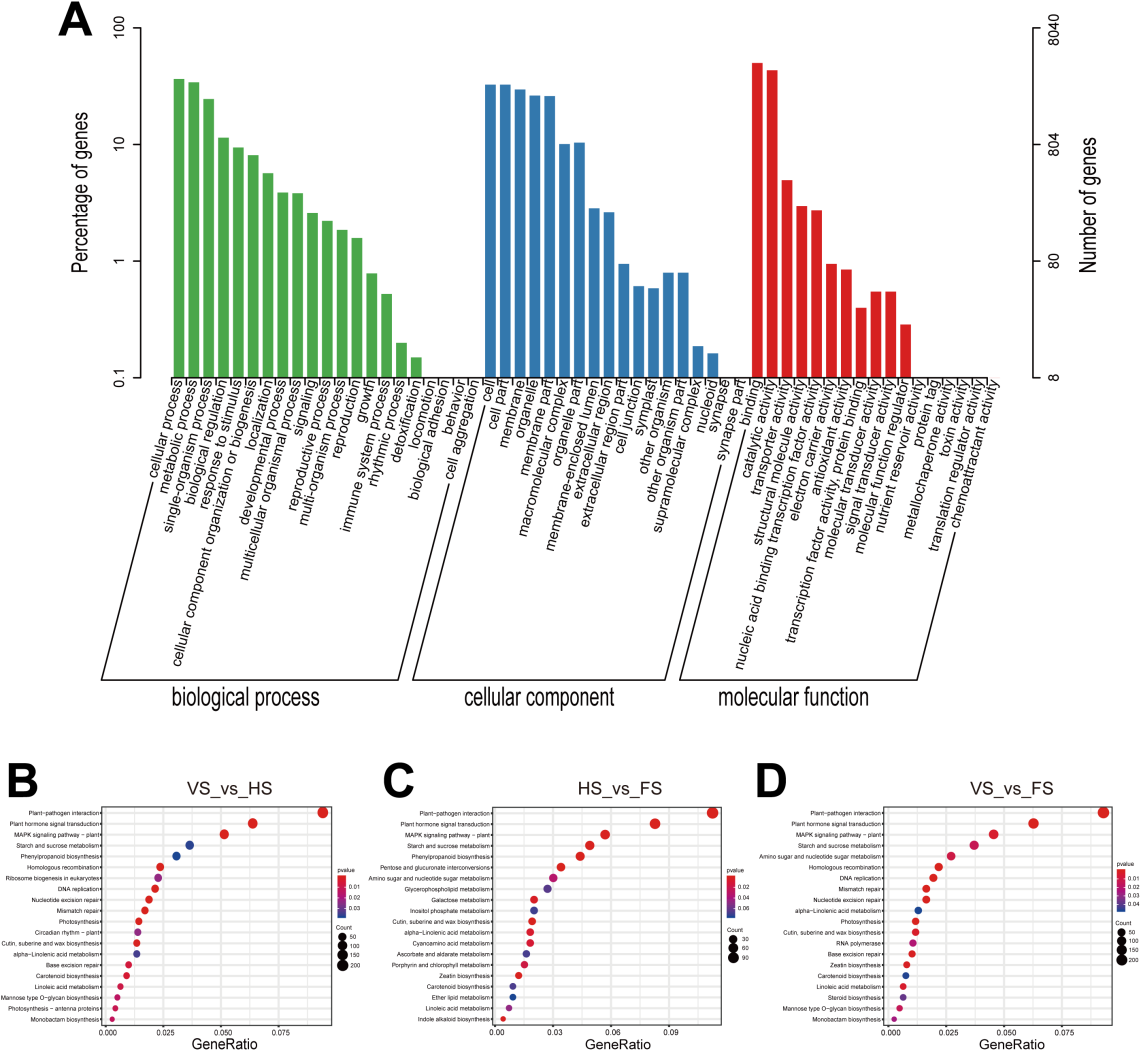
The GO classification and KEGG pathway enrichment of identified DEGs in *E*. *triticeum*. **(A)** GO classification of DEGs. **(B)** KEGG enrichment bubble plot of DEGs in the VS_vs_HS comparison. **(C)** KEGG enrichment bubble plot of DEGs in HS_vs_FS comparison. **(D)** KEGG enrichment bubble plot of DEGs in the VS_vs_FS comparison. The x-axis represents the gene ratio and the y-axis represents the pathways. The size of the bubbles indicates the number of genes enriched in each KEGG pathway. Larger bubbles represent more genes. The color of each bubble indicates the level of significance. VS, vegetative stage; HS, heading stage; FS, flowering stage.

GO enrichment analysis was used to determine the function of DEGs, and three main categories were identified, including molecular function, biological process, and cellular components (**Figure 3A**). A total of 28 functional GO terms were significantly enriched (*P* ≤ 0.05) in different biological processes (**Supplementary Table 4**). The most enriched molecular functions were protein kinase activity (GO: 0004672) and ATP binding (GO: 0005524). In the biological process category, macromolecule modification (GO: 0043412) and regulation of transcription (GO: 0006355) were significantly enriched. The most significantly enriched cellular component terms were integral component of membrane (GO: 0016021) and chloroplast thylakoid membrane (GO: 0009535).

The KEGG pathway enrichment analysis was further used to understand the expression of the DEGs in metabolic pathways, and the *p*-values ≤ 0.05 were considered as a significant enrichment. The top 20 most enriched KEGG pathways are shown in **Figure 3B-D** and **Supplementary Table 5A-C**. The DEGs were mainly enriched in pathways such as plant-pathogen interaction, plant hormone signal transduction, MAPK signaling pathway, starch and sucrose metabolism, phenylpropanoid biosynthesis, pentose and glucuronate interconversion, amino sugar and nucleotide sugar metabolism.

### Identification of the DEGs related to flowering

**Figure 4:**
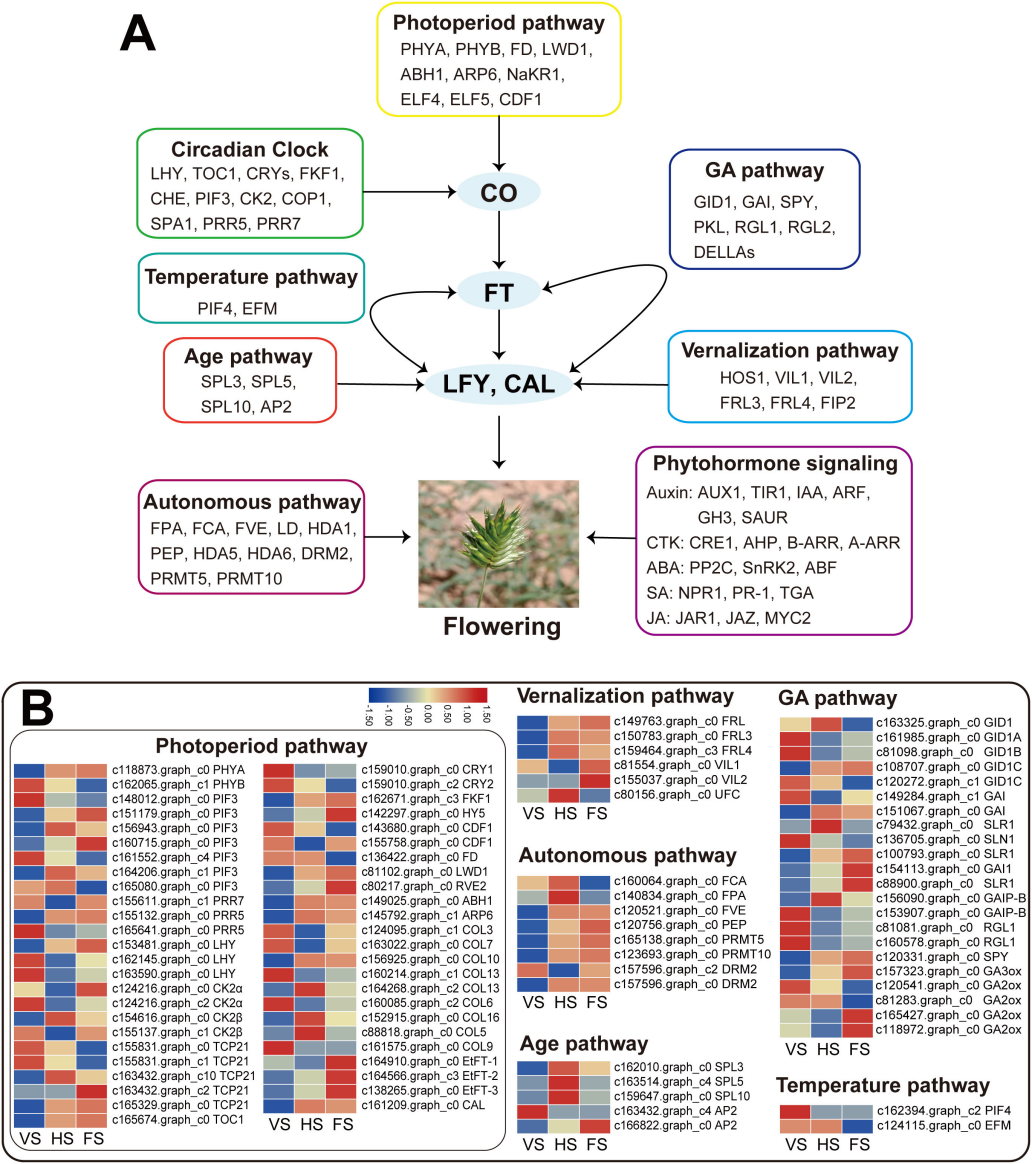
The major flowering pathways in *E*. *triticeum*. **(A)** A putative model of the flowering regulatory network in *E*. *triticeum*. **(B)** The heatmaps of the expression patterns of differentially expressed genes (DEGs) related to flowering in three developmental stages: vegetative stage (VS), heading stage (HS), and flowering stage (FS).

In this study, six major flowering pathways, namely photoperiod (plant circadian rhythm), vernalization, autonomous, aging, temperature, and GA pathways, were analyzed based on transcriptome data. The expression variations of genes associated with these flowering pathways were then compared across three developmental stages. Based on the GO and KEGG annotations of the DEGs, 188 candidate genes related to flowering regulation pathways were initially identified. Among them, 92 DEGs involved in flowering transition were selected (**Supplementary Table 6**). The results showed that most of the DEGs were associated with the photoperiod pathway (48 DEGs), followed by the GA pathway (22 DEGs), the autonomous pathway (8 DEGs), the vernalization pathway (6 DEGs), the aging pathway (5 DEGs), and the temperature pathway (2 DEGs) (**Figure 4A**).

In the *E. triticeum* RNA-seq data, several important genes in the photoperiod and circadian rhythm pathway were identified. These include *RVE2* (*REVEILLE 2*), *PIF3* (phytochrome-interacting factor 3), *LATE ELONGATED HYPOCOTYL* (*LHY*), *TCP*, *HY5*, *COL*, and *TIMING OF CAB EXPRESSION 1* (*TOC1*). These genes were found to be up-regulated in the flowering stage compared to the heading stage. Furthermore, four *FT-like* genes were identified in the three developmental stages, and three of them, including *EtFT-1* (c164910.graph_c0), *EtFT-2* (c164566.graph_c3), and *EtFT-3* (c138265.graph_c0), showed significant up-regulation in the flowering stage (**Figure 4B**, **Supplementary Table 6A**). Given that the *COL* gene is an important core regulator in the flowering pathway, we investigated the evolutionary relationship of the *EtCOL* gene in *E. triticeum* with those in other plants. Phylogenetic tree analysis revealed that the *EtCOL* genes are closely related to the *COL* gene family in wheat and barley (**Supplementary Figure 2A**). The floral meristem identity genes *AP1* and *FRUITFUL* (*FUL*) were not detected in the *E. triticeum* RNA-seq data. However, an *AP1* paralog, the *CAULIFLOWER* (*CAL*) gene, was identified and was found up-regulated at both the head and flowering stages (**Figure 4**, **Supplementary Table 6A, B**). In addition, the floral organ identity gene *LEAFY* (*LFY*) was identified, but no differential expression was observed in the *E. triticeum* transcriptome data (**Supplementary Table 6A**).

In the vernalization pathway, two VIN3-like (VIL) genes showed differential expression, with significantly higher expression levels observed at the flowering stage. In addition, *FRIGIDA-like* (*FRL*) genes (c149763.graph_c0, c150783.graph_c0, and c159464.graph_c0), which were identified as floral repressors that play a crucial role in regulating flowering time in *Arabidopsis* (Wang et al., 2006), were found to be up-regulated at the flowering stage compared to the heading stage. Phylogenetic analysis revealed that two *EtFRL*s were closely related to the wheat *TaFRL*s, while the third showed high homology to the maize *ZmFRL1* (**Supplementary Figure 2B**). These results suggest that *EtFRL*s may be involved in the regulation of vernalization-induced flowering in *E. triticeum*. Notably, the key regulator of vernalization, *FLC*, which plays a central role in *Arabidopsis*, was not detected in the *E. triticeum* transcriptome data, suggesting potential differences in the vernalization pathway between the two species. Similarly, the negative regulator *VRN2*, known to be involved in wheat vernalization, was not detected in the *E. triticeum* transcriptome.

In addition, several putative genes associated with the GA, autonomous, aging, and temperature pathways were identified in the RNA-seq data (**Figure 4B**, **Supplementary Table 6**). In the GA pathway, genes encoding *GAI1* (*GA-insensitive 1*), *SLR1* (*slender rice 1*), *GA2ox* (*gibberellin 2-beta-dioxygenase*), and *GA3ox* were up-regulated in the flowering stage compared to the heading and vegetative stages.

In the aging pathway, transcriptomic analysis revealed key regulators of floral development in *E. triticeum*, including orthologs of *SQUAMOSA PROMOTER BINDING PROTEIN-LIKE* (*SPL*) and *APETALA2* (*AP2*). The *SPL* gene family, which is critical for floral organ growth and fertility regulation through age-dependent pathways (Chao et al., 2017; Xing et al., 2010), showed stage-specific expression patterns: *SPL3*, *SPL5*, and *SPL10* showed the highest expression levels at the heading stage. Phylogenetic analysis revealed that *EtSPL3* was closely clustered with wheat *TaSPL3*, and *EtSPL5* was closely clustered with wheat *TaSPL5* (**Supplementary Figure 3C**), suggesting a similar role in wheat flowering.

In addition, an *AP2* homolog (c166822.graph_c0), which is involved in spikelet determinacy and the transition to floral meristem development (Chuck et al., 2002; Komatsu et al., 2003), was found to be significantly up-regulated at the flowering stage. These results suggest that *AP2* may play a critical role in modulating floral meristem identity and shaping inflorescence architecture in *E. triticeum*.

### Identification of hormone-related DEGs involved in flowering

A total of 197 DEGs in the plant hormone signaling pathway (ko04075) were identified by KEGG pathway analysis (**Supplementary Table 7**). These genes include *AUX1*, *TRANSPORT INHIBITOR RESPONSE 1* (*TIR1*), gibberellin responsive receptor (*GID1*), abscisic acid responsive element binding factor (*ABF*), and ethylene responsive receptor (*ETR*). These genes are involved in the gibberellin (GA), abscisic acid (ABA), auxin, brassinosteroid (BR), ethylene, gibberellin (GA), jasmonic acid (JA), and salicylic acid (SA) signaling pathways (**Table 1**).

**Table 1:** The identified DEGs involved in hormone signal transduction pathways in *E. triticeum*.

Gene entry	Gene name	Signaling pathway	Annotation
K13946	*AUX1*	Auxin	Auxin influx carrier
K14485	*TIR1*		Transport inhibitor response 1
K14484	*IAA*		Auxin-responsive protein IAA
K14486	*ARF*		Auxin response factor
K14487	*GH3*		Auxin-responsive GH3 family protein
K14488	*SAUR*		SAUR-like auxin-responsive protein
K14489	*CRE1*	Cytokinin	Cytokinin receptor
K14490	*AHP*		Histidine-containing phosphotransfer peotein
K14491	*B-ARR*		two-component response regulator ARR-B family
K14492	*A-ARR*		Two-component response regulator ARR-A family
K14493	*GID1*	Gibberellin	Gibberellin receptor 1
K14494	*DELLA*		DELLA protein
K14496	*PYL*	Abscisic acid	Abscisic acid receptor PYL
K14497	*PP2C*		Probable protein phosphatase 2C
K14498	*SnRK2*		Serine-threonine-protein kinase SnRK2
K14432	*ABF*		ABA responsive element binding factor
K14509	*ETR*	Ethylene	Ethylene receptor
K14510	*CTR1*		Serine/threonine-protein kinase CTR1
K13413	*SIMKK*		Mitogen-activated protein kinase kinase 4/5
K14515	*EBF1/2*		EIN3-binding F-box protein
K14514	*EIN3*		Ethylene-insensitive protein 3
K13416	*BAK1*	Brassinosteriod	Brassinosteroid insensitive 1-associated receptor kinase 1
K13415	*BRI1*		Protein brassinosteroid insensitive 1
K14500	*BSK*		BR-signaling kinase
K14503	*BZR1/2*		BZR1 protein
K14505	*CYCD3*		Cyclin D3, plant
K14506	*JAR1*	Jasmonic acid	JAR1 protein
K13464	*JAZ*		JAZ protein
K13422	*MYC2*		bHLH transcription factor MYC2
K14508	*NPR1*	Salicylic acid	NPR1 protein
K14431	*TGA*		Transcription factor TGA
K13449	*PR-1*		Pathogenesis-related protein 1

**Figure 5:**
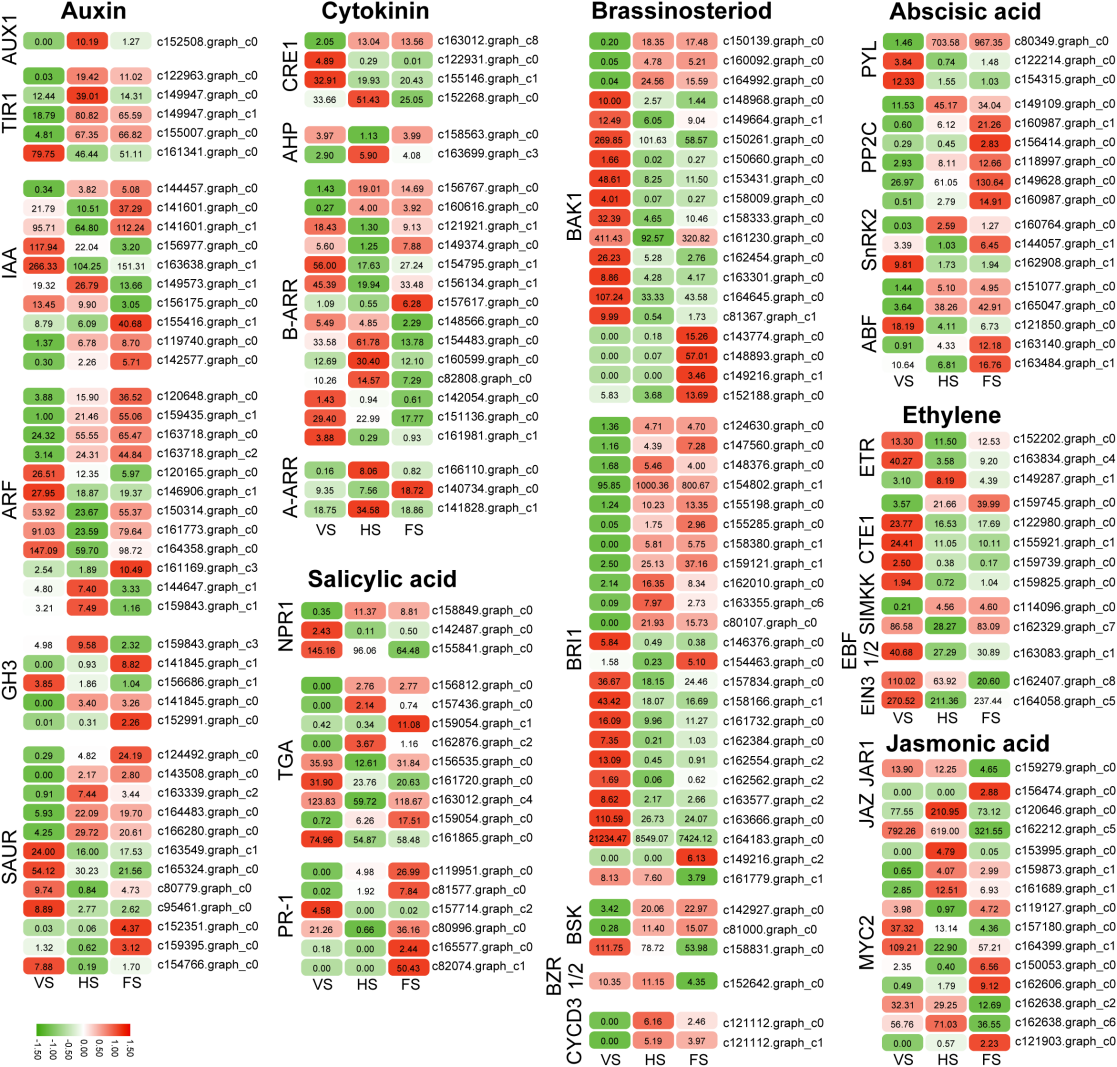
Expression patterns of the differentially expressed genes (DEGs) in the plant hormone signaling pathways.

In the auxin signaling pathway, six genes encoding indoleacetic acid (IAA), five genes encoding auxin response factor (ARF), three genes encoding Gretchen Hagen 3 (GH3), and six genes encoding small auxin-up RNA (SAUR) were upregulated at the flowering stage. In the ABA signaling pathway, four *PP2C*, two *ABF*, and one *SnRK2* were up-regulated at the flowering stage. In the BR pathway, *BAK1* and *BRI1* genes were up-regulated at the flowering stage. Salicylic acid (SA), a hormone signaling molecule, plays an important role in plant immunity and stress response. SA is also involved in the regulation of flowering time. SA regulates flowering time in plants such as *Arabidopsis* by participating in the regulation of the expression of flowering-related genes such as *CO*, *FLC*, *FT*, *FLD*, etc. in the vernalization and photoperiodic pathways (Martínez et al., 2004). Disease progression-related (PR) proteins are induced in plants by the application of various biotic stresses (e.g., pathogens) and abiotic stresses (e.g., drought, cold, salinity, heavy metals, etc.). In this study, PR-1 was found to be significantly increased during the flowering stage of *E. triticeum*, suggesting that it plays an important role in flowering and resistance response in *E. triticeum*. In addition, the genes *TGA* in the SA pathway and *ARR* in the GA pathway were upregulated at the flowering stage (**Figure 5**). These results suggest that the accumulation of phytohormones and hormone-related transcripts is significantly modulated during the flowering stage of *E. triticeum*.

### Identification of flowering pathway-related transcription factors

**Figure 6:**
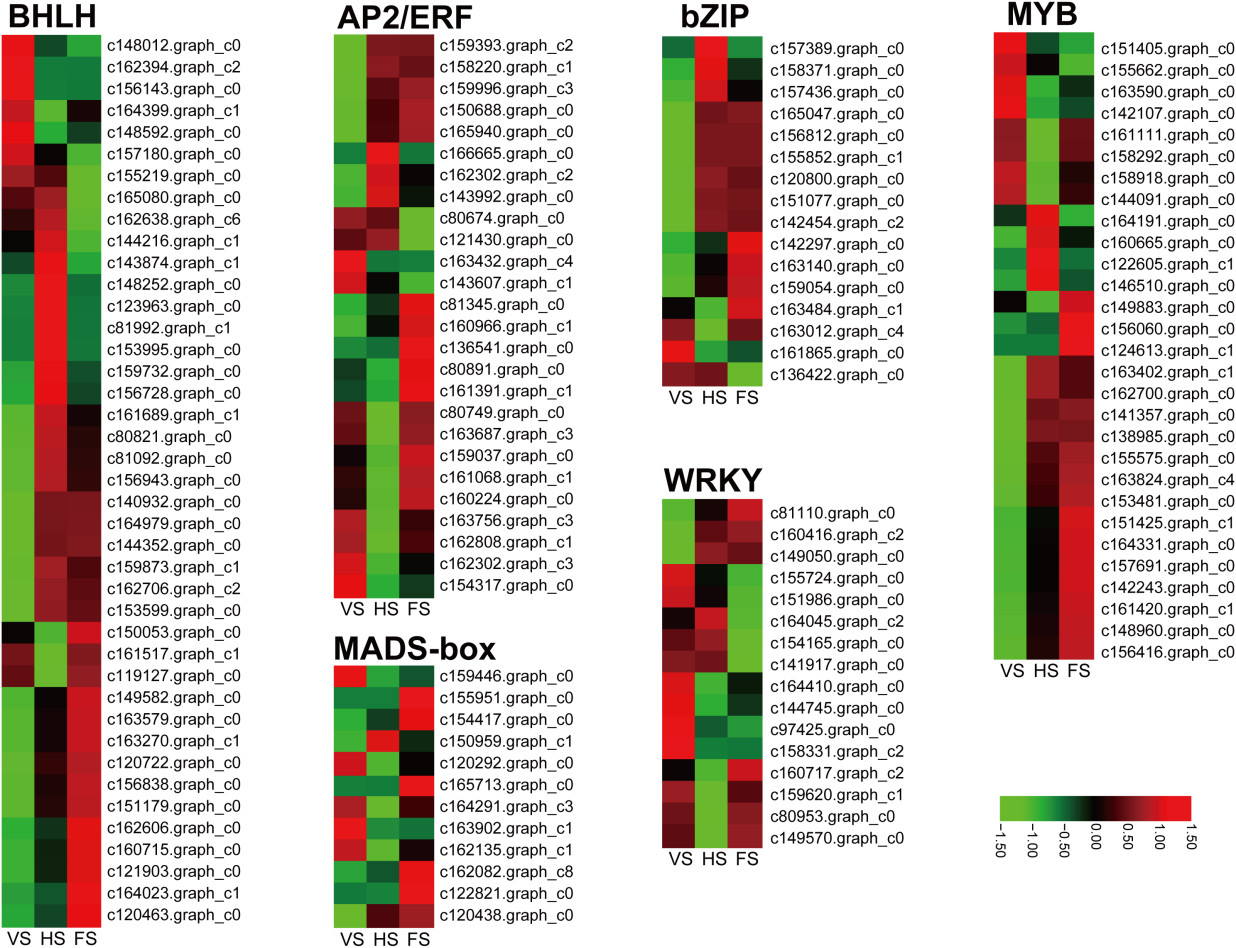
Differential expression of transcription factors at three developmental stages in *E. triticeum*. Red and green represent high and low expression levels, respectively.

In this study, a total of 410 DEGs belonging to 49 transcription factor (TF) families were identified, including *bHLH*, *MYB*, *AP2*/*ERF*, *WRKY*, *bZIP*, *C2C2*, and *MADS*-box. We then detected the expression levels of these genes in the RNA-seq data during the three developmental stages in *E. triticeum*. Notably, a number of TF families exhibited high expression levels at the head or flowering stage. For example, 11 DEGs belonging to the bHLH family were found to be significantly up-regulated at the FS stage compared to the VS and HS stages (**Figure 6**). In addition, several WRKY and MYB transcription factors were identified as significantly upregulated at the FS stage, suggesting a potential regulatory role for these TFs in the flowering process of *E. triticeum*.

MADS-box TFs play critical roles in inflorescence meristem and flower development (Ohmori et al., 2009; Gao et al., 2010). In this study, a total of 12 putative MADS-box genes were identified from the *E. triticeum* transcriptome data, and six genes were predominantly expressed in the flowering stage compared to the vegetative and heading stages (**Figure 6**). To better understand the functions of the *E. triticeum* MADS-box genes, we constructed a phylogenetic tree using 98 MADS-box protein sequences from *Arabidopsis* and wheat (**Supplementary Figure 3**). The results indicated that the *E. triticeum* MADS box could be divided into two groups: M-type (5 genes, 3 Mα and 2 Mγ) and MIKC-type (7 genes). Among the MIKC-type *MADS* box genes, the *Arabidopsis* and wheat orthologs of *AG*, *AGL12*, *SOC*, *TT16*, *SVP*, and *AP3* were identified in *E. triticeum*. Gene expression patterns revealed that two *TT16* (c122821.graph_c0, and c165713.graph_c0), one *AG* (c162082.graph_c8), and one *AP3* (120438.graph_c0) were up-regulated at the flowering stage (**Figure 6**). These results suggest that these TFs may play an essential role in the regulation of flowering in *E. triticeum*.

### qRT-PCR validation

**Figure 7:**
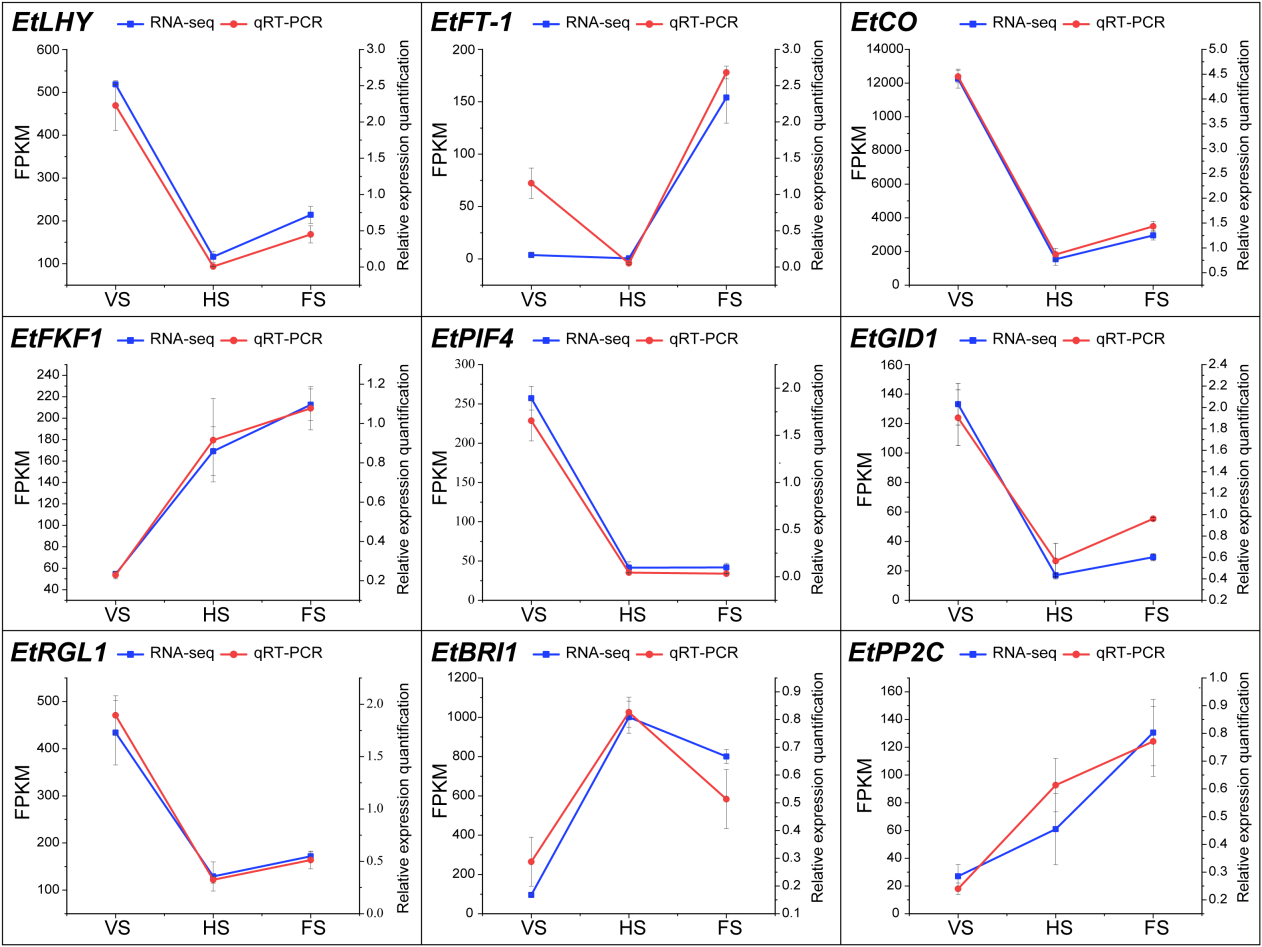
Expression validation of genes between qRT-PCR and RNA-seq at different developmental stages in *E. triticeum*. Red and blue represent qRT-PCR and RNA-seq data, respectively.

To validate the accuracy of the transcriptome results, nine genes were selected for qRT-PCR analysis, including seven genes related to growth and flowering regulation and two genes related to hormone signaling (**Figure 7**). The results showed that the *EtFT-1*, *EtFKF1*, and *EtPP2C* were significantly up-regulated at the flowering stage. These genes have been shown to be important for the circadian clock and the regulation of photoperiodic flowering in *Arabidopsis* (Nelson et al., 2000). Overall, the expression trends of these genes were highly consistent with the RNA-seq.

### Identification and expression pattern analysis of *EtFT* genes

**Figure 8:**
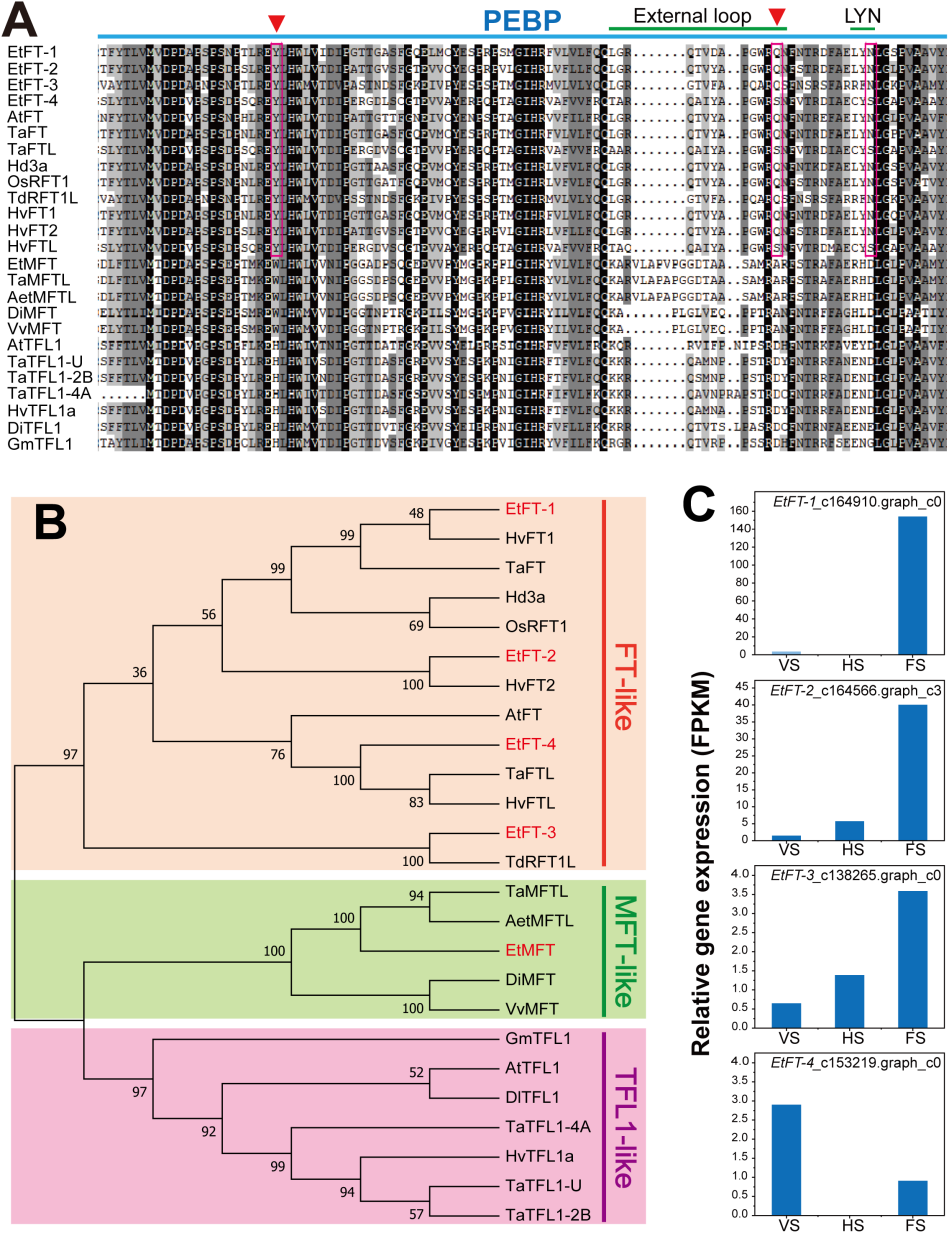
Amino acid alignment, phylogenetic tree and expression analysis of *EtFTs*. **(A)** Multiple sequence alignment of EtFTs from *E*. *triticeum*, along with FT, TFL and MFT proteins from different plant species. AtFT (*Arabidopsis thaliana*, At1g65480); TdRFT1L (*Triticum dicoccoides*, XP_037476013); Hd3a (*Oryza sativa*, AB052941); OsRFT1 (*Oryza sativa*, NP_001056859); TaFT (*Triticum aestivum*, AAW23034); TaFTL (*Triticum aestivum*, XP_044320677.1); HvFT1 (*Hordeum vulgare*, AAZ38709); HvFT2 (*Hordeum vulgare*, ABB99414); HvFTL (*Hordeum vulgare*, XP_044970677.1); TaMFTL (*Triticum aestivum*, XP_044432627.1); AetMFTL (*Aegilops tauschii*, XP_020153395.1); DiMFT (*Dimocarpus longan*, AUG98253); VvMFT (*Vitis vinifera*, NP_001267935); TaTFL1-U (*Triticum aestivum*, TraesCSU02G202000.1); TaTFL1-2B (*Triticum aestivum*, TraesCS2B02G310700.1); TaTFL1-4A (*Triticum aestivum*, TraesCS4A02G409200.1); HvTFL1a (*Hordeum vulgare*, HORVU2Hr1G072750.1); GmTFL1 (*Glycine max*, FJ573238); AtTFL1 (*Arabidopsis thaliana*, At5g03840); DlTFL1 (*Dimocarpus longan*, AHY24028). **(B)** Phylogenetic analysis of four *EtFT* encoding proteins: EtFT-1 (c164910.graph_c0), EtFT-2 (c164566.graph_c3), EtFT-3 (c138265.graph_c0), and EtFT-4 (c153219.graph_c0)) with FT, TFL, and MFT from different plant species. The phylogenetic tree was generated by MEGA 7 software using the neighbor-joining (NJ) method, and the percentage of replicate trees from the bootstrap test (1000 replicates) is indicated beside the branches. Red, green and blue branches represent FT-like, MFT-like, and TFL1-like protein, respectively. **(C)** The relative expression levels of *EtFT-1*, *EtFT-2*, *EtFT-3* and *EtFT-4* genes at three different stages of *E*. *triticeum*. Sample labels are as follows: VS, vegetative stage; HS, heading stage; FS, flowering stage.

The FT and TFL1 subfamilies have undergone functional divergence in flowering regulation, with FT activating and TFL1 repressing flowering, respectively (Wang et al., 2015). In this study, four *FT-like* genes, namely *EtFT-1*, *EtFT-2*, *EtFT-3*, and *EtFT-4*, were identified in the transcriptomic data of *E. triticeum*. Sequence alignment revealed that the EtFT proteins contain a conserved PEBP domain (**Figure 8A**). The conserved residues Tyr85 (Y) and Gln140 (Q) in *Arabidopsis* FT were identified in EtFT-1, EtFT-2, and EtFT-3, which are crucial for FT activity and diagnostic for differentiating FT from TFL1 (Hanzawa et al., 2005; Ahn et al., 2006). However, in EtFT-4, the conserved residue Gln140 (Q) was replaced by Ser140 (S), which is similar to *Triticum aestivum* TaFTL (XP_044320677.1) and *Hordeum vulgare* HvFTL (XP_044970677.1) (**Figure 8A**). In addition, the EtFT proteins contained a conserved outer loop structure (LGRQTVYAPGWRQN) in segment B and the LYN/IYN triad in segment C, corresponding to a potential ligand binding site in FT/TFL1 family proteins (Ahn et al., 2006).

To further investigate the evolutionary relationships of the PEBP family, a phylogenetic tree of EtFT proteins with other plant species was constructed using the neighbor-joining (NJ) method. The results indicated that EtFT-1 was more closely related to *Triticum aestivum* TaFT and *Hordeum vulgare* HvFT1, EtFT-2 was closely related to HvFT2, and EtFT-3 was clustered with *Triticum dicoccoides* TdRFT1L (**Figure 8B**).

To predict the biological function of *EtFT* genes, we analyzed the dynamic expression patterns of *EtFT* genes in the transcriptional data of *E. triticeum*. The results showed that *EtFT-1*, *EtFT-2*, and *EtFT-3* were all significantly up-regulated in the spikelets at the flowering stage. Among them, *EtFT-1* showed the highest expression at the flowering stage compared to the other *EtFT* genes (**Figure 8C**), suggesting its critical role in the regulation of flowering in *E. triticeum*.

### Expression patterns of *EtFT-1* under different day length

Previous studies have shown that *FT* expression is responsive to day length. For example, in *Arabidopsis*, *FT* mRNA accumulates only under long days (LDs) (Suárez-López et al., 2001), whereas rice *Hd3a*, a homolog of *FT*, accumulates mRNA under short days (SDs) (Izawa et al., 2002; Kojima et al., 2002). To investigate whether the accumulation of *EtFT-1* mRNAs follows a similar pattern related to photoperiod-induced flowering, the levels of *EtFT-1* mRNAs were examined under LDs (16 h light/8 h dark) and SDs (8 h light/16 h dark).

**Figure 9:**
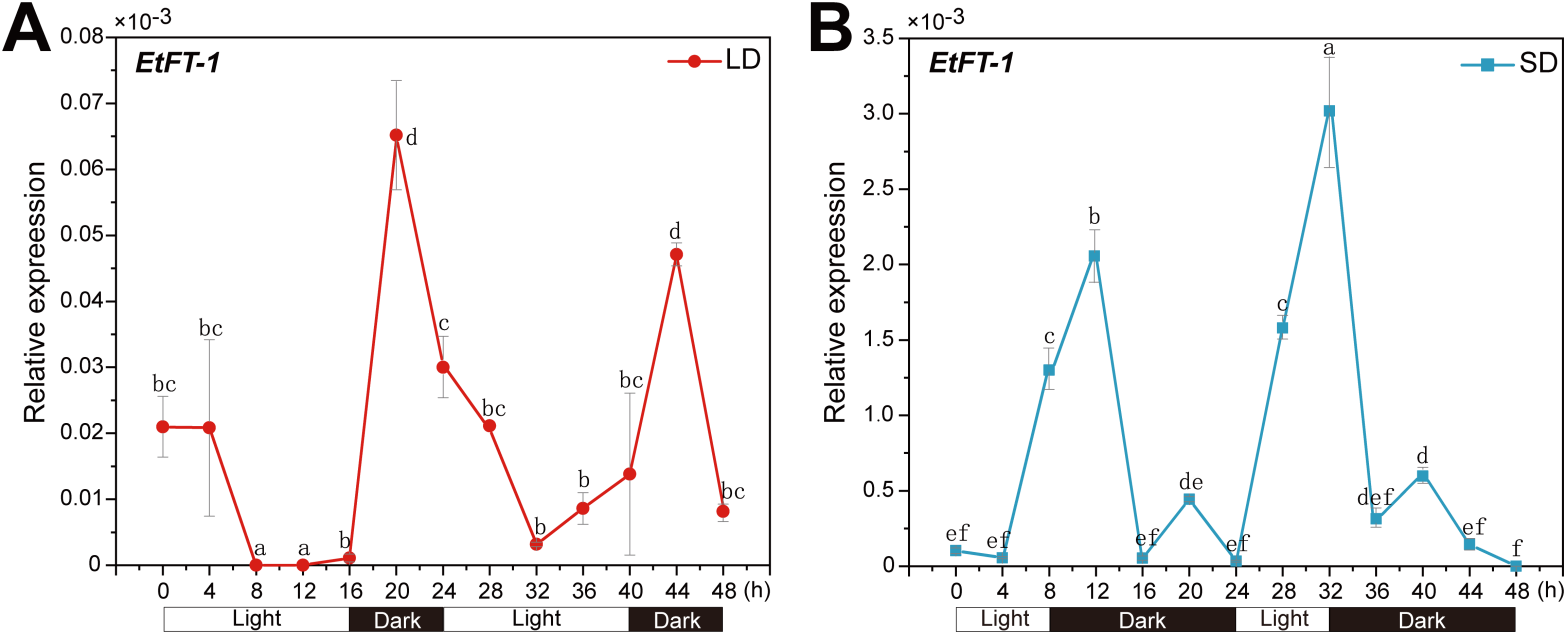
Expression of *EtFT-1* under different photoperiods. Transcript levels of *EtFT-1* in the leaves of 3-week-old *E*. *triticeum* plants grown under long-day (LD, 16 h light/8 h dark) or short-day (SD, 8 h light/16 h dark) conditions by qRT-PCR, with three biological replicates. The 18S rRNA gene of *E*. *triticeum* (GenBank accession No. PQ072787) served as the internal control. Relative expression levels were evaluated by the 2^−ΔCt^ method. **(A)** Expression of *EtFT-1* under LD conditions. **(B)** Expression of *EtFT-1* under SD conditions. Different letters (a, b, c, etc.) indicate significant differences between groups (P < 0.05), while the same letter indicates non-significant differences between groups.

The *EtFT-1* mRNA exhibited a diurnal rhythm in both LD and SD-grown *E. triticeum* plants. *EtFT* expression underwent a more pronounced induction under SD than under LD conditions (**Figure 9**). When plants were exposed to LD conditions, the *EtFT-1* showed a low expression level. However, the transcript level of *EtFT-1* was increased after shifting to darkness and peaked after 4 h of dark treatment (**Figure 9A**). When plants were exposed to SD conditions, *EtFT-1* mRNA showed a pronounced peak around the time of the onset of night (**Figure 9B**). These results demonstrate that unlike *FT* in *Arabidopsis*, *EtFT-1* expression is likely induced in darkness, and short-day conditions promote its expression.

### Overexpression of *EtFT-1* accelerates flowering in *Arabidopsis*

**Figure 10:**
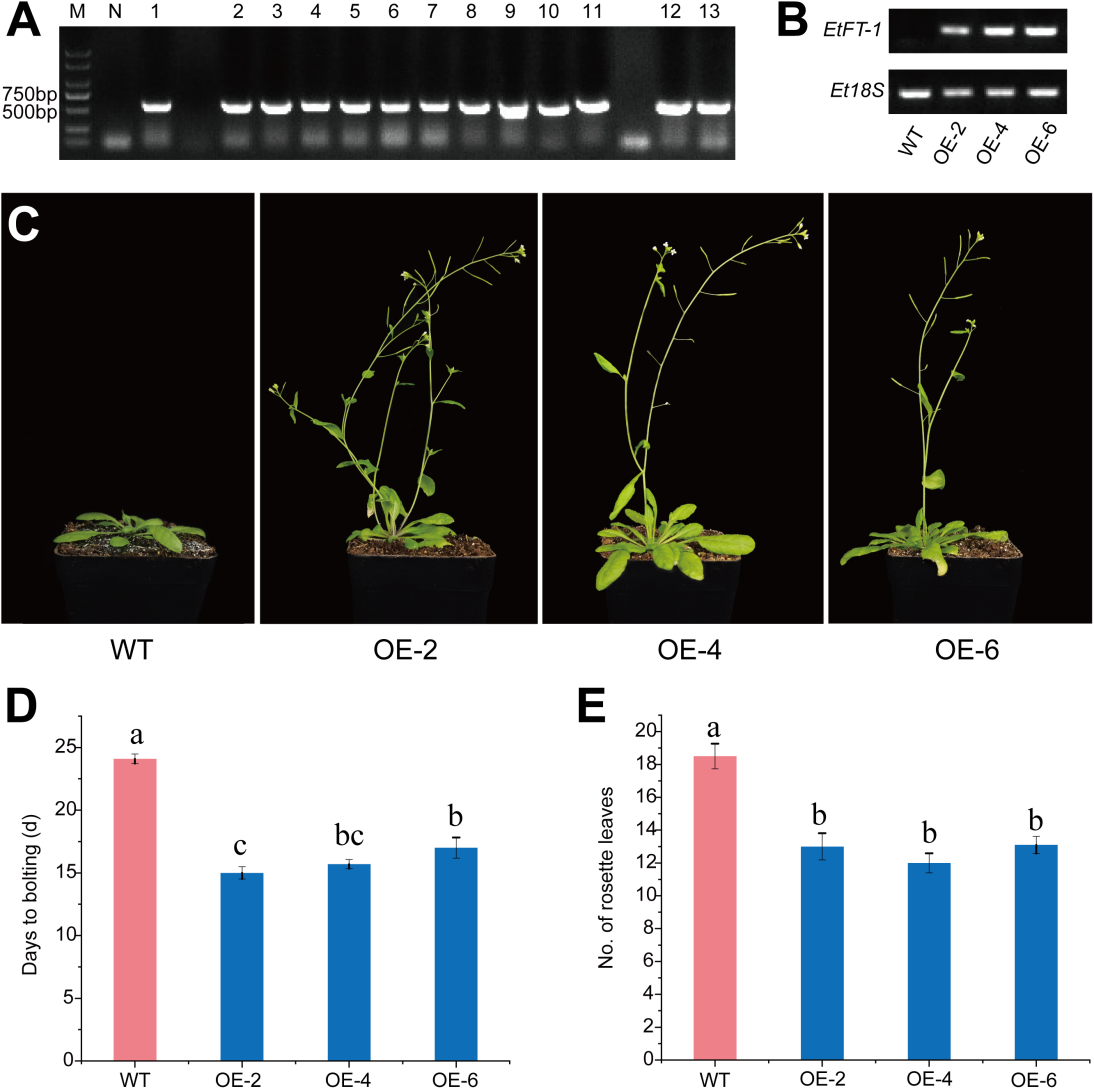
Flowering phenotypes of *EtFT-1* overexpressing lines under 16 h light/8 h dark photoperiod. **(A)** Resistance screening and PCR identification of transgenic *A. thaliana*. M: Marker 2000; N: Negative, template is ddH_2_O; 1-13: PCR identification of transgenic *A. thaliana*. **(B)** Semiquantitative RT-PCR analysis of *EtFT-1* overexpressing *Arabidopsis* lines (OE-2, OE-4, and OE-6) and wild-type (WT) plants. **(C)** The flowering phenotypes of *EtFT-1* overexpressing lines under a 16 h light/8 h dark photoperiod. **(D)** The bolting time in WT and transgenic plants grown under a 16 h light/8 h dark photoperiod. **(E)** The number of rosette leaves of WT and transgenic plants at the bolting stage under a 16 h light/8 h dark photoperiod. Different letters (a, b, c, etc.) indicate significant differences between groups (P < 0.05), while the same letter indicates non-significant differences between groups.

To study the effect of *EtFT-1* on promoting flowering in *Arabidopsis* transgenic plants, the *EtFT-1* overexpression vector driven by the CaMV 35S promoter was constructed and introduced into *Arabidopsis* (Col-0) by *Agrobacterium*-mediated transformation. Homozygous transgenic *Arabidopsis* lines carrying the *EtFT-1* gene were confirmed based on their resistance to kanamycin. Phenotypic analysis was performed on these homozygous plants of the T3 generation, using wild-type (WT) *Arabidopsis* as a control. Genomic DNA was extracted from individual homozygous *EtFT-1* transgenic *Arabidopsis* and WT plants. The target *EtFT-1* gene was then amplified by PCR using specific primers for molecular identification of the transgenic lines (**Figure 10A**). Semi-quantitative RT-PCR was performed to assess the expression of *EtFT-1* in the transgenic lines, and three independent lines with higher *EtFT-1* expression (OE-2, OE-4, and OE-6) were selected for further functional analysis (**Figure 10B**).

The results showed that the *EtFT-1* overexpressing lines exhibited earlier bolting (by 6–10 days) and flowering (by 9–10 days) compared to WT plants (**Figures 10C, D**, **Supplementary Table 8**). In addition, the number of rosette leaves was significantly reduced in the *EtFT-1* overexpressing lines compared to WT plants (**Figure 10E**). These results indicate that ectopic overexpression of *EtFT-1* promotes early flowering in *Arabidopsis*.

## Discussion

### DEGs associated with flowering in *E. triticeum* are mainly related to the photoperiod and vernalization pathways

In the plant life cycle, floral transition has been extensively studied in the model plants *Arabidopsis* and rice in recent years. However, the molecular mechanisms of floral transition in desert ephemerals remain poorly understood*. E. triticeu*m is a widely distributed annual ephemeral species in northern Xinjiang, especially in the Junggar Desert. In extreme desert environments, *E. triticeum* is capable of rapid growth, flowering, and reproduction, completing its life cycle in about two months to avoid severe summer drought conditions. However, little is known about the molecular mechanisms of the floral transition in *E. triticeum*, largely due to the limited genomic data available in public databases. RNA-seq is a powerful tool that has been used to discover novel transcripts and gene regulatory networks. It has become a valuable research tool in biological studies. In this study, 92 flowering-related DEGs were identified (**Figure 4**; **Supplementary Table 6**). These genes were mainly associated with photoperiod, vernalization, gibberellin, autonomous, and age pathways. Notably, several homologs were identified that function as key activators and transcription factors related to flowering in *E. triticeum*, such as the *EtFT* gene family in the photoperiod pathway, the *EtFRL* family in the vernalization pathway, and the *EtAP2* family in the aging pathway.

The influence of the photoperiodic pathway on flowering time is closely linked to circadian rhythm. In many annual plants, flowering time is influenced by the duration of daily light exposure. In *Arabidopsis*, long days promote flowering, while short days inhibit flowering. The *COL* gene, a downstream gene in the photoperiod pathway, controls the transition to flowering by transducing the light signal to the flowering integrator FT (Imaizumi, 2010). Both COL and FT are important mediators in the photoperiodic flowering pathway in *Arabidopsis*. Under long-day conditions, *AtCO* stimulates *FT* expression in leaves (Wong et al., 2013). In rice, the *CO* homolog *Hd1* promotes *FT* expression under short days, but suppresses *Hd3a* transcription under long days, thereby delaying flowering (Kojima et al., 2002). *COL* gene expression is regulated by the circadian clock, and the clock component GI contributes to the formation of the GI-FKF1 complex, which is triggered by light to upregulate *CO* expression under long days (Sawa et al., 2007; Yang et al., 2024). In this study, we found that most of the flowering-related genes in *E. triticeum* are associated with the photoperiod pathway, suggesting its central role in regulating flowering in *E. triticeum*. However, the detailed regulatory mechanism requires further investigation.

Previous studies have shown that the molecular mechanisms of flowering transition are conserved to some extent between the dicot *Arabidopsis* and the monocot wheat, although clear differences remain. For example, the key *Arabidopsis* vernalization regulator gene *FLC* has not been identified in wheat (Milec et al., 2014; Xu and Chong, 2018). In wheat, vernalization is regulated by the *VRN1*, *VRN2*, and *VRN3* genes. The wheat *VRN1* gene is not related to *Arabidopsis VRN1*, but is homologous to the *Arabidopsis AP1*/*CAL*/*FUL* genes (Blümel et al., 2015). VRN2, a CCT protein, has no *Arabidopsis* homologue and acts as a negative regulator of wheat flowering (Yan et al., 2004). The wheat *VRN3* gene is an ortholog of *FT* and promotes flowering in a manner similar to *FT* in *Arabidopsis* (Yan et al., 2006). In this study, we identified a *VRN1* gene homolog, the *CAL* gene, in the *E. triticeum* RNA-seq data, and it was significantly up-regulated at the flowering stage (**Figure 4**). This suggests that it may play an important role in the vernalization induction of *E. triticeum*. However, the negative regulator of wheat vernalization, *VRN2*, was not detected in the *E. triticeum* transcriptome, possibly due to its non-expression or low expression in the tissues tested. This suggests that the vernalization-mediated flowering pathway in *E. triticeum* may differ from that in wheat. Further research is needed to fully elucidate this mechanism.

### Complex transcriptional regulatory network of flowering in *E. triticeum*

An increasing number of studies have shown that several transcription factor families, including bHLH, MYB, Dof, bZIP, WRKY, and MADS-box, play essential roles in regulating the flowering transition (Waseem et al., 2019; Song et al., 2024; Zhang et al., 2020; Zhou et al., 2019a). In this study, 49 transcription factor families were identified from the *E. triticeum* transcriptome data, with many members of the bHLH, MYB, and MADS-box families showing up-regulated expression at the flowering stage (**Figure 6**). MYB FTs play an important role in plant development, participating in various biological processes such as stress and protective responses, seed and flower development (Zhou et al., 2019b). For example, overexpression of the wheat MYB transcription factor *TaMYB72* in rice reduced flowering time by 12 days under long-day conditions (Zhang et al., 2016). In addition, members of the bHLH and MADS-box gene families are essential for plant reproductive development. For example, the *Arabidopsis cib2* mutant exhibits an early flowering phenotype, and ectopic expression of the pineapple bHLH transcription factor *AcCIB2* in the *Arabidopsis Atcib2* mutant can complement the phenotype, indicating that *AcCIB2* shares a similar biological function with *AtCIB2* (Aslam et al., 2020). Similarly, ectopic overexpression of the *PeMADS5* gene from *Phyllostachys edulis* resulted in early flowering and abnormal flower phenotypes in *Arabidopsis* (Zhang et al., 2018). We found that most of the bHLH, MYB, and MADS-box transcription factor genes in *E. triticeum* were highly up-regulated at the flowering stage (**Figure 6**), suggesting that these transcription factors play important roles in regulating flowering in *E. triticeum*.

These transcription factors are known to play important roles in plant responses to abiotic stresses. For example, bHLH transcription factors have been implicated in the regulation of iron homeostasis and stress responses in plants (Qian et al., 2021). Similarly, MYB transcription factors are involved in various stress responses (Li et al., 2019), in addition to the seed and flower development (Chopy et al., 2023). MADS-box transcription factors are critical for reproductive development and can interact with bHLH proteins to regulate floral organ formation. In addition, certain MADS-box genes are involved in responses to abiotic stresses such as salinity, drought, and cold (Di Marzo et al., 2020; Castelán-Muñoz et al., 2019). The up-regulated expression of these transcription factors during flowering may enhance the ability of *E. triticeum* to adapt to the arid desert environment by optimizing reproductive success before the onset of drought.

### Phytohormones regulating flower development in *E. triticeum*

Flowering is controlled by a complex network of genes that work together to make sure it happens at the right time. Hormones, signaling, and internal balance are all important parts of this process (Campos-Rivero et al., 2017). KEGG enrichment analysis of the DEGs showed significant enrichment of plant hormone biosynthesis and signaling pathways during the flowering process in *E. triticeum* (**Figure 5**). These results were consistent with those observed in *Camellia sasanqua* and *Mikania micrantha* (Fan et al., 2022; Liang et al., 2023), suggesting that phytohormone biosynthesis and signal transduction play an important role in regulating the floral transition in *E. triticeum*.

Gibberellins (GAs) are essential for regulating several processes of plant growth and development, including seed germination, stem elongation, and the timing of flowering. In the established GA signaling pathway, GA binds to the soluble GA receptor GID1, which then interacts with the DELLA repressor protein. In petunia, three *GID1* genes, namely *PhGID1A*, *PhGID1B*, and *PhGID1C*, have been identified, and their suppression by virus-induced gene silencing (VIGS) results in reduced growth and delayed flowering (Liang et al., 2014). In the present study, five putative *EtGID1* genes were identified in *E. triticeum*, with the *EtGID1C* gene (c108707.graph_c0) showing high expression levels during the flowering stage (**Figure 4**). This finding suggests that the *EtGID1C* gene plays a role in the flowering process of *E. triticeum*.

Previous studies have shown that SA can affect flowering in several plants, including *Sinningia* sp*eciosa*, *Lemna gibba*, and *Gazania rigens* (Martín-Mex et al., 2015; Fu et al., 2020; Abdul Kareem and Saeed, 2020). In some species, SA is essential for the induction of flowering (Wada and Takeno, 2010). For example, stress-induced SA accumulation promotes earlier flowering in *Arabidopsis* and *Lemna paucicostata* (Martínez et al., 2004; Shimakawa et al., 2012). The SA signaling pathway genes *PR-1* and *TGA* have been implicated in the regulation of floral organ development and flowering (Luo et al., 2022). For example, the *tga7* mutant in *Arabidopsis* showed a delayed flowering phenotype under both long-day (LD) and short-day (SD) conditions (Xu et al., 2021). In this study, we found that most of the *EtTGA* and *EtPR-1* genes were up-regulated at the flowering stage (**Figure 5**), suggesting that the SA pathway may play a regulatory role in flowering in *E. triticeum*.

### *EtFT-1* is involved in flowering time regulation in *E. triticeum*

The FLOWERING LOCUS T (FT) protein, a conserved florigen in angiosperms, integrates signals from multiple flowering pathways (e.g. photoperiod, vernalization) and is translocated from leaves to shoot apical meristems to initiate floral transition (Liu et al., 2021). Loss of *FT* gene function results in delayed flowering under long-day (LD) conditions, whereas its overexpression results in exceptionally early flowering regardless of day length (Kardailsky et al., 1999; Kobayashi et al., 1999). For example, overexpression of the *Arabidopsis AtFT* gene has been shown to promote early flowering in species such as cotton, apple, and poplar (McGarry et al., 2013; Yamagishi et al., 2011; Zhang et al., 2010).

In *E. triticeum*, we identified four *FT* homologs (*EtFT-1, 2, 3*, and *4*), with *EtFT-1* exhibiting the highest expression level during the flowering stage, suggesting its central role in flower induction. To investigate the function of *EtFT-1*, *EtFT-1* was overexpressed in *Arabidopsis*, resulting in transgenic lines with significantly earlier bolting and flowering times compared to wild-type plants. However, the number of the rosette leaves in transgenic plants was reduced, which is a key feature in determining flowering time in *Arabidopsis* (Andrés and Coupland, 2012). These results suggest that *EtFT-1* may plays a critical role in promoting flowering in *E. triticeum*.

Comparative research on a range of plant species has shown that FT homologs are always essential for controlling flowering timing. In rice, *FT-like* genes such as *Hd3a* and *RFT1* are essential for flowering induction under different photoperiods (day and night lengths) (Komiya et al., 2009). Similarly, six *FT* homologs have been identified in soybean (*Glycine max*), with *GmFT2a* significantly promoting flowering under short-day conditions (Sun et al., 2011; Zhao et al., 2016). Four *FT* homologous genes are found in tobacco. While overexpression of *NtFT1*, *NtFT2*, and *NtFT3* delays flowering, overexpression of *NtFT4* promotes flowering (Harig et al., 2012). These investigations demonstrate the evolutionary conservation and functional diversity of *FT* homologs in controlling flowering in a range of plant species.

In this study, we characterized *EtFT-1* through transgenic expression in *Arabidopsis* and confirmed its function in promoting flowering. The roles of the other *EtFT* homologs in the regulation of flowering, however, have yet to be clarified. Further studies should more closely examine the functional divergence of *EtFT* genes using transgenic methods in *Arabidopsis* and monocot species that are more closely related to *E. triticeum*, such as wheat. Furthermore, clarification of their regulatory networks, interacting proteins, and reactions to environmental signals like drought and photoperiod will offer a more profound understanding of the molecular mechanisms that control flowering in desert-adapted ephemerals.

## Conclusion

In this research, RNA-seq was used to investigate gene expression patterns associated with heading and flowering in *E. triticeum*, revealing specific gene networks and key regulators. Transcripts associated with major flowering pathways, including photoperiod, vernalization, GA, and autonomous pathways, were identified, highlighting the importance of genes such as *FT*, *FD*, *COL*, *SPL*, *GID*, and transcription factors from the *AP2*, *bHLH*, and *MADS-box* families. In particular, we identified a novel *FT-like gene*, *EtFT-1*, which promotes flowering when overexpressed in *Arabidopsis*. These findings provide valuable insights into the molecular mechanisms of flowering in *E. triticeum*, and the identified homologs provide a useful resource for plant breeding to regulate flowering time.

## Data Availability

All the data in this study are included in this manuscript. The raw RNA-seq reads have been deposited in the NCBI database (accession PRJNA1198393).

## Supplementary material

Supplementary Figure 1 The NR classification description. **(A, B)** and **(C)** represent the e-value distribution, similarity distribution and species distribution of NR annotations, respectively.

Supplementary Figure 2 Phylogenetic analysis of COL **(A)**, FRL **(B)** and SPL **(C)** proteins in *E. triticeum* and other plant species.

Supplementary Figure 3 Phylogenetic relationship of MADS-box genes in *E. triticeum* (Et), *Triticum aestivum* (Traes), and *Arabidopsis thaliana* (At).

Supplementary Table 1 Summary for the transcriptome assembly.

Supplementary Table 2 Statistics of the transcriptome database of *E. triticeum.*

Supplementary Table 3 Summary statistics of functional annotation of the unigenes of *E. triticeum* in public databases.

Supplementary Table 4 GO enrichment analysis for differentially expressed transcripts with corrected P-valuess ≤ 0.05.

Supplementary Table 5 KEGG pathway enrichment results with upregulated DEGs from the VS_vs_HS **(A)**, HS_vs_FS **(B)**, and VS_vs_FS **(C)** comparison.

Supplementary Table 6 Differentially expressed genes and the related flowering pathway in *E. triticeum*. **(A)** All candidate differentially expressed genes (DEGs) related to flowering in *E. triticeum*. **(B)** Differentially expressed genes associated with the flowering pathway in *E. triticeum*.

Supplementary Table 7 The differentially expressed genes (DEGs) involved in hormone signal transduction pathway in *E. triticeum*.

Supplementary Table 8 Flowering phenotypic analysis of wild-type (WT) and *EtFT-1* overexpressing transgenic *Arabidopsis thaliana* plants.

Supplementary Table 9 Primers used in this study.
