# Supplementary figures and images for: Transcriptome analyses reveal the flowering regulatory networks in the desert ephemeral plant Eremopyrum triticeum
Source: Front Plant Sci. 2025 May 8;16:1576519. doi: 10.3389/fpls.2025.1576519 (PMC12095374; doi:10.3389/fpls.2025.1576519)

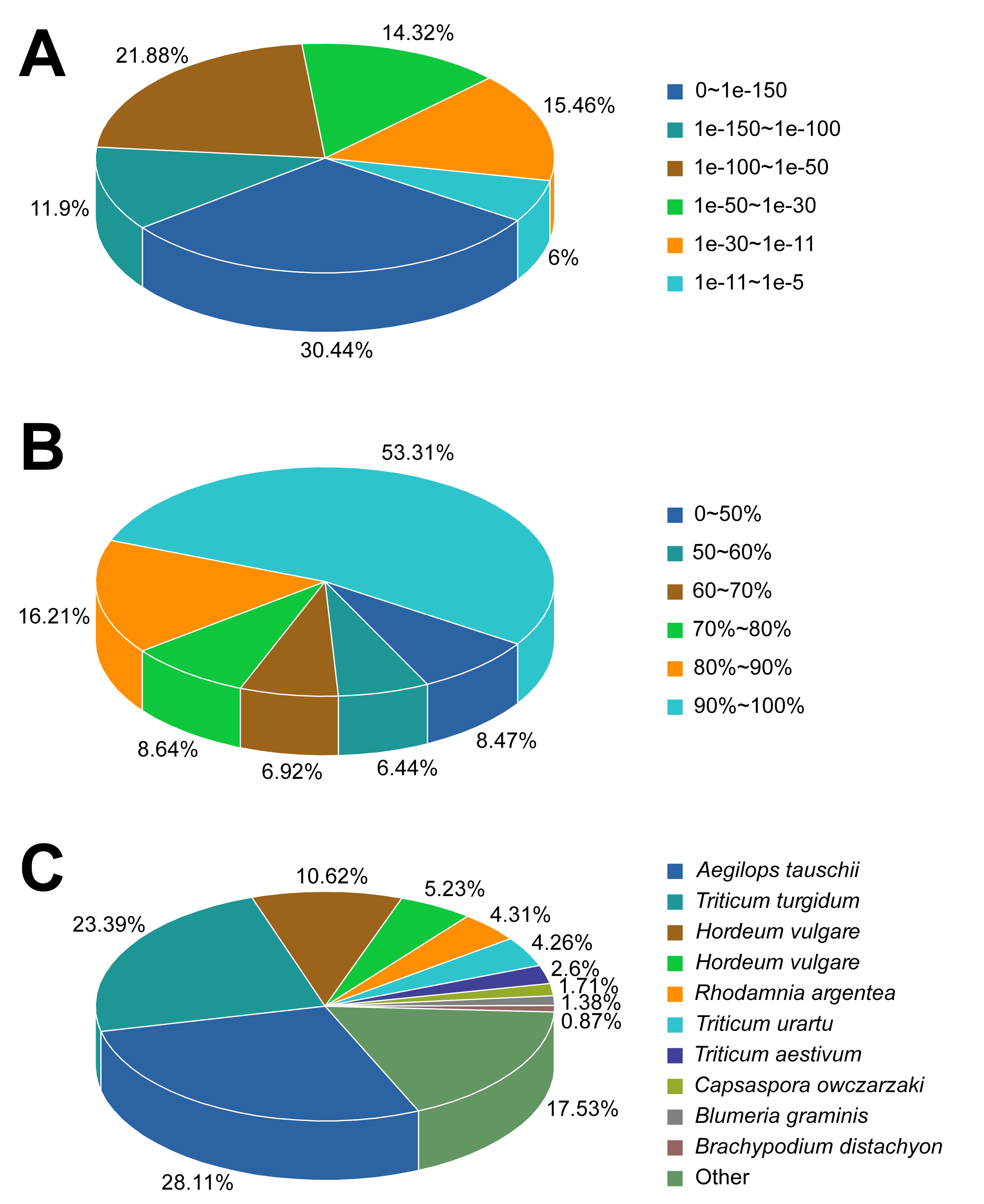

Supplement: Supplementary Figure 1 — The NR classification description. (A, B) and (C) represent the e-value distribution, similarity distribution and species distribution of NR annotations, respectively. [file Image1.tif]

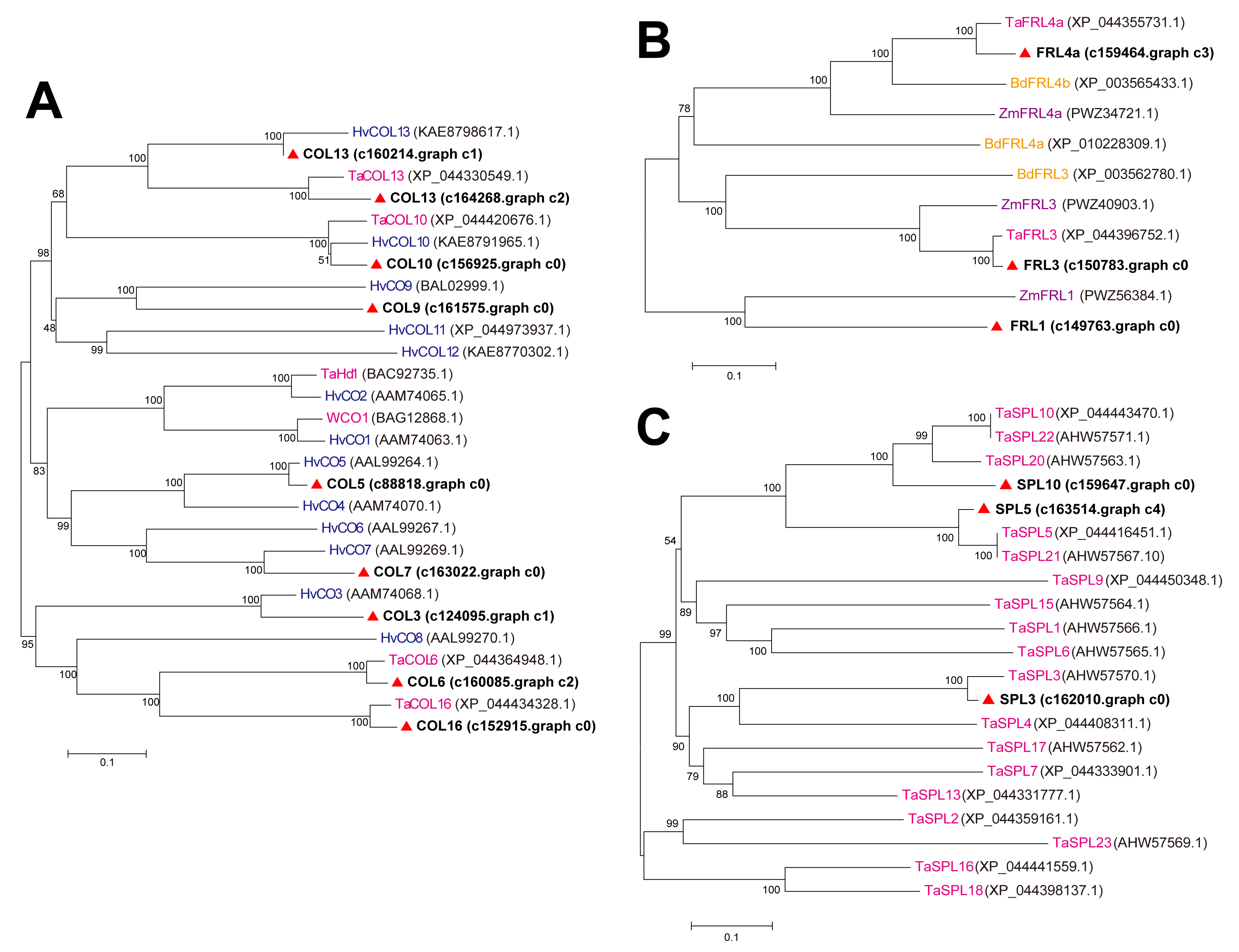

Supplement: Supplementary Figure 2 — Phylogenetic analysis of COL (A), FRL (B) and SPL (C) proteins in E. triticeum and other plant species. [file Image2.tif]

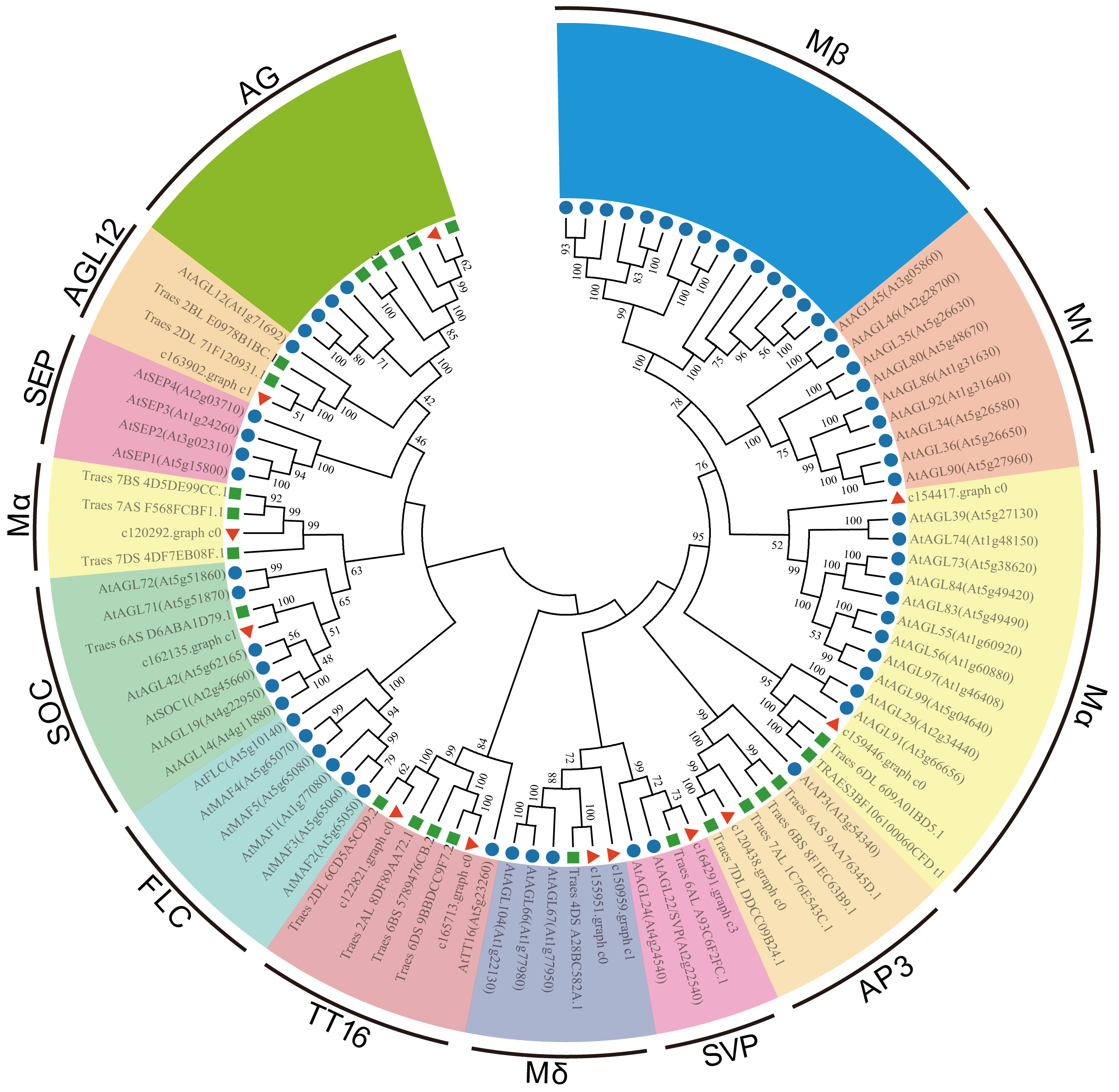

Supplement: Supplementary Figure 3 — Phylogenetic relationship of MADS-box genes in E. triticeum (Et), Triticum aestivum (Traes), and Arabidopsis thaliana (At). [file Image3.tif]
